# Perceptions and Attitudes Toward an Interactive Voice Response Tool (Call for Life Uganda) Providing Adherence Support and Health Information to HIV-Positive Ugandans: Qualitative Study

**DOI:** 10.2196/36829

**Published:** 2022-12-06

**Authors:** Phoebe Kajubi, Rosalind Parkes-Ratanshi, Adelline Twimukye, Agnes Bwanika Naggirinya, Maria Sarah Nabaggala, Agnes Kiragga, Barbara Castelnuovo, Rachel King

**Affiliations:** 1 Infectious Diseases Institute College of Health Sciences Makerere University Kampala Uganda; 2 Department of Public Health & Primary Care, Institute of Public Health University of Cambridge Cambridge United Kingdom; 3 Department of Internal Medicine, College of Health Sciences, School of Medicine Makerere University Kampala Uganda; 4 Institute for Global Health Sciences, University of California, San Francisco San Francisco, CA United States

**Keywords:** mobile health, mHealth, mobile communication technologies, people living with HIV, antiretroviral therapy, quality of life, Uganda

## Abstract

**Background:**

The continuing decline in AIDS-related deaths in the African region is largely driven by the steady scale-up of antiretroviral therapy. However, there are challenges to retaining people living with HIV on treatment. Call for Life Uganda (CFLU) is an interactive voice response tool using simple analogue phones. CFLU supports patients with daily pill reminders, preappointment reminders, symptom reporting and management, and weekly health promotion tips. Mobile health tools are being increasingly used in resource-limited settings but are often adopted without rigorous evaluation.

**Objective:**

This qualitative study conducted at 12 months after enrollment assessed patients’ experiences, perceptions, and attitudes regarding CLFU.

**Methods:**

We conducted a qualitative substudy within an open-label randomized controlled trial titled “Improving outcomes in HIV patients using mobile phone based interactive software support.” Data were collected through 6 focus group discussions with participants sampled based on proportion of calls responded to—<25%, between 25% and 50%, and >50%—conducted at the Infectious Diseases Institute, Mulago, and the Kasangati Health Centre IV. NVivo (version 11; QSR International) was used in the management of the data and in the coding of the emerging themes. The data were then analyzed using content thematic analysis.

**Results:**

There was consensus across all groups that they had more positive than negative experiences with the CFLU system. Participants who responded to >50% of the calls reported more frequent use of the specific elements of the CFLU tool and, consequently, experienced more benefits from the system than those who responded to calls less frequently. Irrespective of calls responded to, participants identified pill reminders as the most important aspect in improved quality of life, followed by health promotion tips. The most common challenge faced was difficulty with the secret personal identification number.

**Conclusions:**

Findings showed participants’ appreciation, high willingness, and interest in the intervention, CFLU, that demonstrated great perceived potential to improve their access to health care; adherence to treatment; health awareness; and, consequently, quality of life.

**Trial Registration:**

ClinicalTrials.gov NCT02953080; https://clinicaltrials.gov/ct2/show/NCT02953080

## Introduction

### Background

The decline in AIDS-related deaths in the African region is largely driven by the steady scale-up of antiretroviral therapy (ART) [1]. Of the 16.3 million (64%) people living with HIV accessing treatment in 2018, a total of 52% had a suppressed viral load [1]. Similarly, in Uganda, a total of 1.16 million of the estimated 1.38 million people living with HIV were on ART by December 2018, giving a coverage of >90% on ART [2]. In addition, evidence shows that there has been remarkable progress toward ensuring that people initiated on ART have their viral load suppressed [2,3]. For example, Uganda reached a viral load suppression rate of 90.4% in 2017 to 2018 [4].

Despite these notable achievements, retention on treatment of people living with HIV is an increasing challenge [5], coupled with challenges of maintaining adherence [5-8]. In 2010, the average rate of early mortality and loss to follow-up in resource-limited countries was estimated at 23% across the sub-Saharan region [9]. Finitsis et al [3] conducted a meta-analysis of 84 observational studies where nearly 40% of participants had <90% adherence.

Amid these challenges, adherence to ART is reportedly influenced by certain factors that differ by region of the world, which include socioeconomic and disease- and ART-related factors as well as factors related to people living with HIV and their families [10,11]. A study on adherence to ART and its determinants conducted among patients infected with HIV in Nigeria revealed that forgetfulness, busy daily tasks, occurrence of adverse effects, and *too many pills to take* constituted the major reasons for missing doses [10]. Consequently, these factors cause poor adherence to therapy, resulting in treatment failure and the development of viral resistance to antiretroviral medications [6,11,12].

In light of this, it is important to develop additional innovative, practical, targeted, and feasible interventions to improve retention and increase and maintain levels of adherence among patients with HIV on ART if treatment failure and resistance is to be avoided to achieve maximal viral load suppression [8,11,12]. Odili et al [10] suggest that interventions to improve adherence to ART should address challenges such as forgetfulness among the patients and frequent occurrence of adverse effects and consider specific patient-related factors such as daily tasks.

In response to this urgent need, the potential of mobile health (mHealth) communication technologies in closing the gaps in the HIV treatment continuum and their use have grown significantly over the years [13-15]. The World Health Organization recommends the use of mobile phone–based technologies for management of chronic diseases and ART adherence [16,17]. The benefits of mHealth technologies in health care have been reported worldwide and recommended as an opportunity to increase the quality and cost-effectiveness of health care, particularly in resource-constrained settings amid the growing number of patients [18-21]. Studies have shown that mobile phones are used throughout lower-income countries more than any other modern technology; have the potential to revolutionize health care, particularly in low-resource settings where health care infrastructure and services are often insufficient; and offer great promise for improving the quality of life [6,15,21-25]. Important benefits of using mHealth technologies highlighted in systematic reviews and other studies include adherence to treatment (for people living with HIV and other categories of patients) being the most significant [6,14,22,26-34], high effectiveness for the dissemination of health promotion messages and lifestyle tips [19,20,22,25,35,36], reminders for physician appointments or improvement in clinic attendance [6,23,25,28,33,37,38], remote diagnosis [39,40], emergency medical response [22,41,42], improvement in communication and information delivery and retrieval processes over vast distances between health care service providers and patients [22], privacy and convenience allowing the user to be in charge of the process [13], and improvement in viral load suppression [28]. However, a major concern raised by some studies regarding SMS text messaging–based interventions is the variability in the magnitude of study outcomes [43,44]. Chib et al [15] observed that the mHealth literature in low- and middle-income countries (LMICs) reveals a growing body of knowledge, yet existing reviews suggest that projects yield mixed results.

### Objectives

We undertook a randomized controlled trial (RCT) in Uganda (trial registration: ClinicalTrials.gov NCT02953080) using an intervention entitled Call for Life Uganda (CFLU), an mHealth tool or software that is based on the open-source Mobile Technology for Community Health (Grameen Foundation), described elsewhere in a separate publication [45]. The RCT hypothesis was that CFLU would increase medication adherence, virological outcomes, and HIV knowledge to give an overall increased quality of life in vulnerable populations starting or established on ART or switching to second-line ART, including special populations such as pregnant women, discordant couples, and young people in Uganda.

This paper describes a qualitative substudy conducted at 12 months after enrollment aimed at assessing *similarities* and *differences* in the experiences, perceptions, and attitudes of people living with HIV regarding the CLFU tool by response rate category.

## Methods

### RCT Study Design

This qualitative study was part of an open-label RCT titled “Improving outcomes in HIV patients using mobile phone based interactive software support” (NCT02953080). The intervention was Call for Life, a software adapted from Connect for Life, and was first piloted in India and Ghana [46,47]. It was developed by the Grameen Foundation and Janssen, the pharmaceutical companies of Johnson and Johnson [47]. CFLU was adapted to support people living with HIV in Uganda in a variety of ways. It interacts with people living with HIV using SMS text messaging or interactive voice response (IVR) functionalities (a technology that allows a computer to interact with humans through the use of voice and tones input via keypad). Participants in the RCT received either the usual standard of care plus the CFLU mHealth tool (intervention arm) or standard of care with clinic support and provider-initiated counseling (control arm). Intervention participants received the usual standard of care plus daily adherence IVR reminders (or SMS text messaging) delivered just before the usual pill-taking time. The system sends a phone call to the participant and, when they answer it, plays music until they enter a personal identification number (PIN) to access further services. They also received preappointment reminders; had access to educational health tips once weekly to increase knowledge of HIV and comorbid conditions; and had an option to call a toll-free line and report symptoms or medication side effects, which would prompt health care workers to call back within 24 hours. The primary objective of the RCT was to determine the effect of the CFLU tool on the quality of life of people living with HIV in urban and semiurban Ugandan health facilities at 6, 12, and 24 months or at closeout of the project [45]. The study was conducted at 2 sites. The first was the Infectious Diseases Institute (IDI), Mulago, a specialist private not-for-profit urban HIV clinic established within Makerere University, located near the National Mulago Hospital Complex serving >8000 people living with HIV. The second study site was Kasangati Health Centre IV (KSG HCIV), a semiurban government-owned clinic that provides comprehensive HIV care and treatment and serves approximately 5000 patients actively undergoing HIV care. It is located in the Wakiso District, which comprises both rural and urban areas and has a population of >2 million people. Eligible patients were randomized into either the control (standard of care) or intervention arm (1:1 ratio) in the RCT. At baseline, participants in the intervention arm were trained on how to initiate and receive calls, and the messages were provided in four languages: English, Luganda, Kiswahili, and Runyankore. A description of how the tool was introduced to the participants is detailed in 2 separate study publications that include the RCT study and the qualitative exploratory study findings [45,48]. At baseline, all patients underwent a quality-of-life assessment, which was repeated at months 6, 12, and 24.

### Qualitative Substudy

Patients enrolled in the intervention arm of the CFLU RCT were purposively sampled to participate in the qualitative substudy. Two lists (one for KSG HCIV and another for IDI Mulago) were generated, categorizing the proportion of calls answered by the patients (ie, patients who had a proportion of <25% of calls answered; those who had a proportion of 25%-50% of calls answered; and, finally, those who had a proportion of >50% of calls answered). Patients from these 3 categories based on the proportion of all calls answered were contacted by telephone. The purpose and study procedures were explained to them in a call, and they were recruited based on their availability to participate in the study.

We used a descriptive qualitative design following a phenomenological research approach to explore lived experiences and perspectives of participants who used the CFLU tool to gain a deeper insight to address the research questions. Specifically, the qualitative study conducted at 12 months after enrollment assessed *similarities* and *differences* in the experiences, perceptions, and attitudes of people living with HIV regarding the CLFU tool by response rate category. Participants were asked to describe their experiences with the CFLU tool, specific elements of the tool that they had used (daily pill reminders, health tips, appointment reminders, symptom reporting, and adherence checks), what they particularly liked about the CFLU tool and why they liked those specific elements, frequency of use, the secret PIN code, what they disliked about the tool, comfortability of use, lessons learned, suggestions on how the tool could help other patients, how the tool could be improved, and patients’ willingness to pay for the CFLU system.

### Data Collection

The qualitative study used focus group discussions (FGDs) as the method of data collection to stimulate a rich discussion. The FGDs were conducted by 2 social scientists, which included a graduate counselor (AT) and a senior social scientist (PK) experienced in qualitative methods of data collection and conversant in Luganda, a language widely spoken at the 2 study sites. They worked as moderator and note-taker for every FGD. The FGDs were conducted following a topic guide. Each FGD had between 5 and 14 participants, lasted between 1 and 1 and a half hours, and was audio recorded with participant permission. The FGDs were scheduled at a time convenient to participants and were conducted at the 2 study sites in venues that allowed for privacy. Confidentiality was maintained by use of a coding system during the FGDs. Participants were told of this anonymity and the measures to be taken. Data were collected using handwritten notes plus audio recordings to capture details of the FGD responses.

### Data Management and Analysis

The recorded FGDs were transcribed and translated from Luganda into English verbatim. The two social scientists (PK and AT) cross-checked and verified the transcripts for consistency and accuracy and carried out preliminary thematic coding through multiple readings of the transcripts. The next step involved PK and AT understanding the data and developing a list of thematic categories for constructing a codebook. The research objectives, FGD topic guide, and transcripts guided the generation of a codebook. There were 7 major codes, namely, positive experiences with the CFLU tool, negative experiences with the CFLU tool, specific elements of the tool that patients used, frequency of calls, comfort of use, lessons learned, and suggestions for improvement. The data set was imported into NVivo (version 11; QSR International) [49] for coding, managing, and retrieving. Once all the transcripts were brought together, systematic coding was undertaken. Codes were assigned to relevant segments of the text; similar codes were aggregated to form themes that were then used to address the research questions and develop coherent narratives [50]. This involved the 2 social scientists, who independently read through the relevant section of a transcript within the NVivo project and pooled together the relevant segments into a node (which is the NVivo equivalent of a theme). Independent codes were compared and discussed to ensure rigor and trustworthiness [51]. Coding helped identify all segments of data that related to the particular node listed in the codebook. In some cases, there were more complex relationships where data were coded into more than one node.

### Ethical Considerations

Ethical clearance was obtained from Makerere University School of Public Health Higher Degrees Research and Ethics Committee (378) and the Uganda National Council for Science and Technology (HS 3005), and the study was registered at ClinicalTrials.gov (NCT02953080). Written informed consent was obtained from the participants for involvement in the main RCT; participation in the study was voluntary based on informed consent. The substudy team were trained to administer informed consent in the language best understood by the participants. Objectives of the study and procedures to be followed during the FGDs were explained to all participants. Written consent for participation in the qualitative study was sought after the participants received the study information, objectives, and procedures. The research assistants (PK and AT) read the consent form out loud, and all participants provided written informed consent. Results were disseminated to participants and stakeholders, which served as a member-check activity.

## Results

### Demographics and Sample Characteristics

A total of 300 participants were enrolled in the intervention arm of the parent RCT, and 256 (85.3%) of them completed the 12-month follow-up [45]. Findings from the parent RCT showed that at 12 months, 9.8% (25/256) of the active participants were low users (0%-24%), 41.8% (107/256) of the participants were moderate users (25%-50%), and 48.4% (124/256) of the participants were high users (>50%).

For the qualitative study, a total of 52 participants (n=19, 37% male and n=33, 63% female) took part in 6 FGDs: 2 (33%) from the KSG HCIV study site and 4 (67%) from the IDI Mulago study site (Table 1). The mean age was 43 years and the overall age range was 22 to 64 years.

**Table 1:** Characteristics of the focus groups.

FGD^a^ category	Study site	Participants, n (%)			Age range (years)
		Male participants	Female participants		
FGD 1—responded to <25% of calls (n=8)	KSG HCIV^b^	0 (0)	8 (100)	23-32	
FGD 2—responded to <25% of calls (n=5)	Infectious Diseases Institute, Mulago	2 (40)	3 (60)	32-38	
FGD 3—responded to 25% to 50% of calls (n=14)	KSG HCIV	4 (29)	10 (71)	22-47	
FGD 4—responded to 25% to 50% of calls (n=9)	Infectious Diseases Institute, Mulago	4 (44)	5 (56)	25-64	
FGD 5—responded to >50% of calls (n=9)	Infectious Diseases Institute, Mulago	9 (100)	0 (0)	34-50	
FGD 6—responded to >50% of calls (n=7)	Infectious Diseases Institute, Mulago	0 (0)	7 (100)	29-56	

^a^FGD: focus group discussion.

^b^KSG HCIV: Kasangati Health Centre IV.

### Participants’ Use and Experiences With the CFLU Tool

#### Overview

Participants were asked what they knew about the CFLU tool and to describe the specific elements of the tool that they had used, their experiences with the tool, and what they particularly liked and disliked about the CFLU system and why they liked those specific elements. Irrespective of proportion of calls responded to, there were more similarities than differences among the 3 categories (ie, those who had a proportion of <25%, 25%-50%, and >50% of calls answered) regarding what participants knew about CFLU, their use, their experiences with the tool, and what they particularly liked about it. Findings revealed that *Pill reminders* were reportedly the most popular and were perceived to be most beneficial among the specific elements of the CFLU tool, followed by health tips, symptom reporting, and appointment reminders.

#### Pill Reminders

##### Overview

Throughout the responses, it was evident that participants across all FGDs largely attributed the positive experiences with the CFLU system first to pill reminders that assisted them in taking their medicines on time, resulting in improved adherence and better health and quality life compared with the health challenges before their involvement with the CFLU system:

Before enrolment, I was very sick; smelling bark cloth (cloth made out of the bark of a tree used to wrap dead bodies ready for burial) i.e. I was so close to death and I was about to be buried in my home village. Then they put me on CFLU. But ever since I joined the system, they remind me to take my medication, educate me about healthy living. From that time, my health has been okay. The virus is even asleep. I feel much better than before so please continue with it.

KSG HCIV, responded to 25%-50% of calls, FGD 3

Across the different categories, there was concurrence in participants’ description of their *medicine-taking practices* before and after enrollment in the CFLU system. Most participants from the 3 categories reported that, before their involvement with the CFLU system, they used to forget to take their medicines on time because of busy work schedules, reluctance to take the pills, pill burden, absence of a person to remind them, and lack of proper food to take with the medicines. However, with the pill reminders, participants reportedly took their medicines on time even amid busy schedules and other challenges:

Before, I would forget, but ever since I started getting pill reminders, I don’t forget however busy I may be; I still remember the time for taking. I work in a hotel so sometimes I may be so busy, but when I see the call, I remember that it is time for taking pills. I had poor adherence and it used to affect me; but now it doesn’t.

KSG HCIV, responded to <25% of calls, FGD 1

Findings further revealed that even participants who responded to <25% of calls used and benefitted from pill reminders. Low responders often had challenges with their PINs or secret PIN codes, which prevented them from having access to other CFLU components. Consequently, most of them felt that CFLU system was just about pill reminders. They explained that, whenever the phones rang, they were unable to access other services but took the call only as a reminder to take their pills:

Yes, reminding me of the time to take my pills. Because there’s nothing much to it; as soon as the phone rings, I know straight away that it is a pill reminder...that very number is for pill reminders. They told us to put in the PIN and talk, but my PIN refused to work, in fact I have never used it.

KSG HCIV, responded <25% of calls, FGD 1

##### Benefits Associated With Pill Reminders

In addition to improved adherence, experiences of improved self-esteem and hope to live positively were topics within all FGDs attributed to the CFLU phone calls that enhanced clients’ psychosocial support. Participants passionately used terms such as “*parent*, *counselor*, *friend*” to refer to CFLU indicating what the system meant to them. Across all FGDs, participants explained that the answering machine spoke very calmly, kindly, and politely to them, making them feel loved, cared for, and accepted:

They don’t ignore you; they show you love and care. For instance, some of us who are HIV positive, we reach a point and cut off relations with people, we get fed up of people. But now, that gentleman who speaks (the voice), he speaks so gently and calmly to me; there’s a way he speaks to you, he puts you in the mood, he makes you feel loved...they have not abandoned us. They have helped us a lot.

KSG HCIV, responded 25%-50% of calls, FGD 3

#### Health Tips and Associated Benefits

##### Overview

In addition to pill reminders, health tips were particularly appreciated and found very useful in raising awareness about health-related information and were linked to improvement in health and quality of life and promoting a healthy lifestyle. Again, this was a topic across all FGDS, especially among those who responded to >50% of calls, followed by those in the 25% to 50% category and, finally, the few among the <25% category who did not have challenges with their secret PIN codes and could access the option for health tips on their phones. Participants mentioned that the educative health tips provided a range of very useful information that they had not been exposed to during previous counseling and educational sessions at health facilities and other forums, which included information on chronic illnesses (such as breast and cervical cancer and diabetes), encouraging them to attend medical checkups for early detection and treatment, and on other diseases, including tuberculosis and sexually transmitted infections such as candidiasis. Other health tips mentioned included information on behavior change such as abstinence, faithfulness to one sexual partner and condom use, positive living, nutrition, family planning, pregnancy, and breastfeeding. Improved self-esteem and boldness to teach others were also attributed to the educative health-related information from the health tips. This was mostly recounted by participants who responded to calls >50% of the time as they listened more to the health tips than the other 2 categories of participants. The former purportedly disclosed their status readily to family members and nonrelatives and practiced positive living:

It has educated us, and has also made us educate others. Because in our villages people still point fingers that so and so is HIV+. But it has helped us be doctors to others; you explain to someone that if they have these signs they need to see a doctor because it may be such and such a disease. In other words, they keep us informed about various diseases that attack people.

IDI Mulago, responded to >50% of calls, FGD 5

They boldly shared the learned health tips with their families and community members; encouraged members of the community to attend HIV counseling and testing and start medication if found HIV-positive; and, for those found HIV-negative, they encouraged them to abstain, be faithful to their partners if married, or practice safe sex using condoms:

What I know about it is, it teaches us about health, diseases which are dangerous and all other ordinary diseases. And they continue to educate you even on what you don’t know because they reach a time and ask you, “do you still want to listen to more health tips?” The benefits are many, I cannot exhaust them all. That is what I like most about it.

IDI Mulago, responded to >50% of calls, FGD 6

Most participants, especially those who responded to calls >50% of the time, requested to be called on weekends particularly to listen to the health tips, some with their family members because this was when they had quality time. Participants pointed out that the CFLU system also gave them the option to listen to the same health tips several times:

This time when I listen to health tips about HIV/AIDS, the next time I listen to cancer health tips, the following time I listen to health tips concerning sexual behaviour. I keep changing because there may be health tips about another disease that they may have brought. I press different health tips one at a time so that I can memorize.

IDI Mulago, responded to >50% of calls, FGD 6

##### Positive and Healthy Living

Participants attributed positive (healthy) living to messages from the health tips, which encouraged them to discard fear about being HIV-positive and imminent death, accept their status, and live positively. Consequently, this helped them have hope in life and lead productive lives:

Another thing, it has helped me at least to come out boldly, and I can confront the fear which I had in me that I am going to die. I know that yes I have HIV but I won’t die, I’ll live longer. The more I take my medication, the more I will live longer by being healthy. Positive living in everything I do.

IDI Mulago, responded to >50% of calls, FGD 6

Nutritional advice encouraged consuming more fruits and vegetables, drinking a lot of water, eating a balanced diet, and having meals on time.

##### Behavioral Change

Participants described aspects of behavior change that they were practicing from the knowledge acquired from the health tips, as illustrated as follows:

What I have put in practice, is condom use. I and my husband are HIV-positive but I don’t want his virus (Laughs) and I also would not want my virus to mix with his. Yes, we have put that in practice. In addition to that, sleeping under treated mosquito nets, I boil my water, I don’t mix local herbs with the medicine. Even these condoms, sometimes we sit in youth groups and I educate them that using a condom does not mean you are HIV+, it means you are simply protecting yourselves.

IDI Mulago, responded to >50% of calls, FGD 6

#### Symptom Reporting and Associated Benefits

Patients were given a toll-free number to report any danger signs, request information, and receive medical advice and emergency assistance. In addition, health workers would call them, inquire about any health-related problem they had, and advise accordingly. Again, this was a topic across all FGDs; participants appreciated the “symptom reporting” component.

Many participants passionately stated that the symptom reporting component demonstrated to them that the *basawo* (health workers) really cared for them and went the extra mile of calling them to find out if they had any health issues. They appreciated the fact that they had someone to talk to anytime they had health problems and that, most of the time, the *basawo* endeavored to call them or follow up with them advising them on what medicines to take or where to buy the medicines or instructed them to go to the health facilities for treatment even when it was not their appointment day. They found this beneficial as sometimes they did not have money for transport to hospitals; other times, such health issues happened when it was not their appointment day. They had trusted and informed people to confide in about their problems and, in most cases, they were helped:

Another thing about CFLU, it has helped me so much in a way that you are like our parents. You call us and ask, “do you have any illness?”...you take the responsibility to call us and give us advice on what to do, or how to get help...I am so happy about it and my health, because if it was not for CFLU, my health would have been really bad.

IDI Mulago, responded to >50% of calls, FGD 6

Consequently, this improved the relationship between the participants and health workers, resulting in increased trust and confidence of the participants in them, which encouraged them to call any time and report health-related problems:

It makes you feel proud that someone cares for you. We used to think you “basawo” (health workers-doctors) do not care for us because people always complain about that. But here we are more than certain that we are cared for and that makes us happy.

IDI Mulago, responded to >50% of calls, FGD 6

By contrast, a few participants, particularly among those in the <25% category, reported challenges that hindered full use of the symptom-reporting component. Some reportedly were not aware of the toll-free number, and some did not know how to use it, as stated as follows:

There is a toll free number for CFLU, I also tried to call it but they said it does not exist on the network. And then I thought to myself, did the doctor give me a wrong number?

IDI Mulago, responded to <25% of calls, FGD 2

Across all categories, many participants complained about the long waiting hours to receive a response from the physician after reporting a health problem.

#### Appointment Reminders and Associated Benefits

Among the CFLU system elements, this was the least used. Most participants did not receive appointment reminders. Of the few who received this service, most reported having received appointment reminders once or twice, and this was mostly among those who responded to >50% of calls, followed by those in the 25% to 50% category and, finally, the few among the <25% category who did not have challenges with their secret PIN codes. Among participants who received appointment reminders, this component was most appreciated by those employed in jobs that involved frequent traveling across long distances and also those in mobile businesses or trade. Participants in this category explained that such jobs kept them so busy that they depended on these reminders to keep their medical appointments:

CFLU has helped me to keep my appointment. They always give me a message that you have an appointment this Tuesday.

IDI Mulago, responded to >50% of calls, FGD 5

### Frequency of Calls and Comfort of Use

At enrollment into the CFLU system, all participants reportedly asked for an appropriate time and day to receive calls. Most reportedly received calls daily, some of them twice daily:

If you take your pills twice a day, in the morning and evening, they call you at those times. But if you take once a day, they’ll also call once.

KSG HCIV, responded to 25%-50% of calls, FGD 3

Furthermore, participants were asked to rate their comfort level with using the CFLU system on a scale of 1 to 5 (5 being most comfortable) and give reasons for the rating. Most rated it 4-5, pointing out that they were comfortable using it and could clearly explain the different options. Other reasons included the training given to use the system, easy-to-follow prompts, expeditious rectification of technical issues, and the confidentiality of the PIN code.

However, participants who had challenges with the secret PIN code could not explain clearly whether they were comfortable using the system.

### Negative Experiences With the CFLU Tool

The study further explored participants’ challenges regarding using the CFLU tool. The number and proportions of mentions of the challenges are summarized in Table 2.

**Table 2:** Number and percentages of responses of negative experiences with the Call for Life Uganda (CFLU) tool (N=52).

Themes and subthemes			Responses, n (%)
**PIN^a^ challenges**			
	Blocked PIN	11 (21)	
	PIN refusing to work	8 (15)	
	Incorrect PIN code	7 (13)	
**Technical issues**			
	Phone ringing endlessly	9 (17)	
	Wrong timing of the pill reminders (calling before or after agreed time)	8 (15)	
	Inconsistent and irregular calls	4 (8)	
	Calling participants from different CFLU system numbers	4 (8)	
	Nonresponse of CFLU system to participants’ calls	3 (6)	
Long waits for physician’s response after reporting a symptom			4 (8)
Challenges with toll-free number			3 (6)

^a^PIN: personal identification number.

Several challenges were reported by participants, most centered on the secret PIN code. Participants narrated challenges they had with the PIN code, most explaining that, even when they reported and received another number, it could not work:

You have to put in the PIN code because that’s the only way they will know whether you took the pills on time or not. I tried it the first time and it refused, I tried it again and they gave me another PIN code which also refused to work. Then they called me and I told the doctor that they refused; then I was told to read the other one, which I did, and I tried it as well but it also didn’t work.

KSG HCIV, responded to <25% of calls, FGD 1

Most participants in this category reportedly resorted to listening only to pill reminders when the attempt with the second PIN code failed. They explained that, whenever the phone rang, they would just know that it was time to take their pills and would not press for other options. These complaints were largely expressed by participants who responded to <25% of calls:

As for me doctor mine refused from the very beginning; even when I came here they told me the system is still being rectified. So I have never spoken to anyone on that call or listening to anything. Most times when I pick up I only hear the kadodi but most times I don’t even pick up the call, I just end it because I already know the essence of the call.

KSG HCIV, responded to <25% of calls, FGD 1

The most reported challenge in this category were blocked PINs when responding to a phone call:

My view about the PIN is that, my PIN was blocked; I was on 2255 and I thought that maybe they found that 2255 is commonly used by many people and they blocked some so that they could get other PINs. But I do not think one can forget their pin code; because I did it for a month and they would keep telling me it’s not the one, I should try again. Until I called the CFL contacts and they told me to get another PIN because it seems the previous one has some issues. But I think they should do it for us; they should get for us the pin codes that we shall use. That’s it.

IDI Mulago, responded to <25% of calls, FGD 2

These were followed by participants whose PIN codes reportedly refused to work, as explained as follows:

My PIN code also refused to work; the one they gave us. So I came back here and was given a new PIN which also refused to work. However much I keep pressing it says the PIN is incorrect. So it just keeps ringing.

KSG HCIV, responded to <25% of calls, FGD 1

Similar to the aforementioned issues were challenges reported by participants who were told repeatedly that their PIN codes were wrong or incorrect even when they received new PINs:

About the pin code, there should be a change, I am not sure if it is us who forget our PINs but I am sure they are the ones; but then they tell me it is incorrect. So I do not know if I am the wrong one or is it the callers that are wrong. So I would like to be helped in that area.

IDI Mulago, responded to <25% of calls, FGD 2

Challenges further included technical issues with the CFLU tool, which comprised the phone ringing endlessly, wrong timing of the pill reminders, inconsistent and irregular pill reminders, calling participants from different CFLU tool numbers, and nonresponse of the CFLU tool to participants’ calls.

Participants who complained about the phone ringing endlessly found this challenge wearisome. They stated that, even when they entered the PIN, the phone would continue ringing without communicating any message:

Doctor sometimes it rings so endlessly. It gets to a point beyond what I can handle; and get fed up and just switch off the phone.

KSG HCIV, responded to <25% of calls, FGD 1

Participants further complained about wrong timing of the pill reminders, that is, being called before or after the agreed time. Participants who faced this challenge strongly believed that it was a CFLU system error:

What I don’t like about it is, I am supposed to get my pill reminder at night, but then at around 11am and I get the call. At first I did not know and when I come I am told to press some things and it would not respond. Then the doctor called me to ask about my health, then I also told her about that issue. I was told, when it calls at a wrong time I just press option 5. That was my only issue. I would wonder why they called at that time yet I am supposed to get the call at night.

IDI Mulago, responded to >50% of calls, FGD 6

Related to the aforementioned challenge, participants further complained about inconsistent and irregular calls whereby, unlike the aforementioned challenge where the timing was inconsistent, participants facing this challenge were called on diverse days, which they found perplexing:

Doctor one more thing; sometimes they take a week without calling. I don’t really know why. You wait for the pill reminders in vain; then after a week they resume.

IDI Mulago, responded to 25%-50% of calls, FGD 4

Participants further complained about the CFLU tool calling them from different numbers, which they found disturbing and confusing. The numbers were reportedly from different countries that included Kenya, Burundi, Sudan, and South Africa. Some recounted that, when the “*kadodi*” played and they entered the password, no information was communicated until the call ended:

I think what they are complaining about is faced by more than one person. Because I personally get calls from 3 different numbers; when you receive all of them; they play the “kadodi” but when you enter the password, it does not give you the information you want; until the call switches itself off. It has now taken 2 weeks when I enter the password and it fails.

IDI Mulago, responded to >50% of calls, FGD 5

The last technical issue participants pointed out was nonresponse of the CFLU tool to participants’ calls. The few participants who experienced this challenge explained that, sometimes, when they called back after receiving a call from the CFLU tool, either the latter would go silent or would not pick up the call:

In addition to what she said, sometimes they may call and you wonder what is happening; then you try to call them back but they don’t answer you. Airtime is spent, and yet you haven’t gotten any response.

IDI Mulago, responded to 25%-50% of calls, FGD 4

Another type of challenge concerned two aspects of CFLU namely, symptom reporting and the toll-free number. A few participants complained about long waits for the physician’s response or never talking to a physician at all after reporting a symptom:

I may have a headache or a fever; on the phone; and they tell me they are going to give me a professional doctor to speak to me; but I have never talked to them or gotten any feedback from the doctor, or telling me to go to hospital. That’s it.

KSG HCIV, responded to <25% of calls, FGD 1

The last negative experience stated by participants was the nonfunctional toll-free number. The few who experienced this challenge complained about not receiving a response when they called the toll-free number, being told that they should not call that number, or receiving a response that the number did not exist on the network:

As for me, I would like to know, if I want to talk to the doctor directly, the toll free number that we were given directly, whenever I call it, no one answers. Unless if it is the counsellor that calls me directly. But if it is me calling directly, it is hard for them to pick up.

IDI Mulago, responded to >50% of calls, FGD 6

### Patients’ Ideas and Suggestions to Improve the CFLU Tool

The study captured patients’ suggestions on how to improve the CFLU tool during the FGDs. The following suggestions emerged from the FGDs on how to improve the CFLU tool.

#### Expanding CFLU to Other Areas, Social Places, and Media and Including More People

This was reportedly the most common suggestion made to improve the CFLU tool. Participants suggested this idea based on the benefits from the tool, most importantly, improvement in quality of life attributed to pill reminders and health tips. Participants considered reminders necessary for all patients, especially those residing in rural areas. They recommended that the tool should be expanded to include other people with a similar condition and not be limited to only urban health facilities but also extended to rural facilities, schools, and churches and extended further to media such as radios and televisions such that, on hearing these messages, affected or infected people would be inspired to get tested for HIV and be started on treatment. Participants were aware of the importance of adherence to medication and felt that CFLU would motivate others to adhere if enrolled. Illustrative quotes are provided in Textbox 1.

**Illustrative quotes from participants regarding expanding Call for Life Uganda.**

**More people should be allowed to join Call for Life Uganda**

- *Doctor, I am suggesting, even those who have not yet joined it, they should put them all on the system because all of us came here to work on our health. Whoever is seeking for good health should join CFL...because it helps to remind them; what brought them was their quest for good health. So when it helps them, it is not bad on their side. They will just be appreciative just like we are here who have already joined it*. [IDI Mulago, responded to 25%-50% of calls, FGD 4]

**Call for Life Uganda should be scaled to rural health facilities**

- *As for me, what I am suggesting is, they should not only have the system in the hospitals within the city; but they should also take the system to rural hospitals and health centers so that they can also benefit...so that they also enjoy a good life; because some people fear coming to these big public hospitals due to self-stigma. So if you also go to those places and educate them as well, one may come to hospital without any problem about it*. [KSG HCIV, responded to 25%-50% of calls, FGD 3]

**The Call for Life Uganda tool should be extended to the media**

- *Doctor; even if it is not rural areas alone; the doctors can come up with something; just like the herbalists; they are always on radios and TVs talking about what they do. Many people watch TVs and listen to radios; someone may be changing stations on the radio and randomly land on those herbalists speaking. So even if the person feels hopeless, they may hear this herbalist teaching about their medicine; and also see doctors teaching the same on TV. I think it would be of good help. Even those that had stopped taking their medicine will start; those that hadn’t gone for testing will go and test themselves*. [KSG HCIV, responded to <25% of calls, FGD 1]

**The Call for Life Uganda tool should also be extended to schools**

- *One more thing, they should also go to schools, talk to the youth about how to prevent themselves from catching HIV/AIDS; in secondary schools, they could do free HIV testing; basically extend their health services to these schools*. [KSG HCIV, responded to <25% of calls, FGD 1]

#### Including Additional Health Tips (Topics) and Providing Detailed Information

This was the second most common suggestion proposed by participants to improve the CFLU tool. Interestingly, even participants who had challenges with the secret PIN code and were not receiving the health tips suggested health tips to include in the CFLU tool. Regarding additional health tips, the following were suggested: nutrition (ie, improved diet that comprises healthy eating and increased consumption of fruits for their nutritional benefits), behavior change (including topics on domestic violence and its effects on health), safe conception for discordant couples (eg, prevention of mother-to-child transmission and sexually transmitted diseases), and sensitization about medications and their effects.

The following is an illustrative statement:

According to me, I think what should be added, is first of all about the fruits. You could say maybe in a week or month, decide that this week we are talking about this fruit. You outline its benefits and all other information concerning it, and this helps to urge more people to consume that fruit. For example, you may say this week you are talking about beetroot, and you go in depth in information concerning this fruit, and then the following week you talk about another fruit. You could also add some information which may not be medical as such. For instance, domestic violence; if it is in a family, even taking medicine changes; the health takes a negative turn, as well as other things. So if you decide that this week you are talking about domestic violence, and then you get good words to use for that. It could even be a case study, and you get a true story which is educative. It will make a person more anxious and anticipate for when they will get the next health tips.

IDI Mulago, responded to 25%-50% of calls, FGD 4

A participant expressed the need for frequent provision of health tips:

About the health tips, what you can add on is maybe call on a daily basis to increase in knowledge; because learning never stops. I am sure that it will really help us to understand more about our health, and what we are following.

IDI Mulago, responded to <25% of calls, FGD 2

Conversely, a participant was of the view that health tips may also be sent as SMS text messages to be read later during free time by patients with busy work schedules, as expressed as follows:

According to me; as a transporter, that is why sometimes I have very little time to look into those tips because I’m always having constant phone calls. Is there anyway those tips can be sent to us who are part of CFL, and we get some of these tips as messages on the phone. So that in your free time you can read through them; because us transporters we are always traveling; from here to Rwanda, Burundi, Congo. We are always on the move so there is hardly any time for us to listen to those health tips. Just like we get messages, WhatsApp messages, depending on your PIN.

IDI Mulago, responded to 25%-50% of calls, FGD 4

#### Providing Appointment Reminders to All Participants

Several participants suggested sending appointment reminders to all those involved with the CFLU tool as it was only a few who reportedly received them. Some suggested that reminders to get their medicine refills should be sent a few days before the appointment date:

In my opinion, they should send the appointment reminders 2 or 3 days to the date of appointment. They can call on Friday and say, “do you know that you have a medical checkup on Monday” “do not forget to go to the hospital.” It would be very good but I have never received it. They should really do it like CFL.

IDI Mulago, responded to >50% of calls, FGD 5

#### Resolving CFLU Tool Technical Issues Faced by Participants

Regarding specific technical issues revolving around the CFLU tool, participants made the suggestions outlined in Textbox 2.

**Illustrative quotes from participants regarding solving technical issues.**

**The Call for Life Uganda tool or system should have the same number (one common number) used to call patients to avoid confusion**

- *What I am saying is; please have a permanent number on which you call us. There are about 3 different numbers that call me; there are numbers that call, you put in the password but the “kadodi” continues ringing. Yet the main number I know, when I put in the password it works very well. But some of those other numbers that call, we do not know them. I saved the CFL main number as “dawa” and as soon as I see it ringing I just put in the password and listen. But when the other numbers call, they reject my password*. [IDI Mulago, responded to >50% of calls, FGD 5]

**Resolve issues of irregular or inconsistent calling times**

- *What I don’t like about CFL, let them make sure if it is 10 o’clock that I chose to get my pill reminders, let them not call me at 1am or 2am. I am kindly requesting; that it should strictly be at 10pm*. [IDI Mulago, responded to <25% of calls, FGD 2]

**Rectify issues of blocked personal identification numbers**

- *In most cases they call me; But I stopped getting the health tips because my pin code was blocked; so that is one of the issues I wanted to address to my doctor so that it can be rectified and I continue getting those health tips; because they help me a lot; when it comes to positive living*. [IDI Mulago, responded to <25% of calls, FGD 2]

**Address the challenges encountered with the toll-free number**

- *One more thing; there is a toll free number for CFL I also tried to call it but they said it does not exist on the network. And then I think to myself; did the doctor give me a wrong number? I also request that they work on that*. [IDI Mulago, responded to <25% of calls, FGD 2]

**Continuation of the Call for Life Uganda intervention even after completion of the study**

- *I request that even when we complete the study you continue giving us calls*. [IDI Mulago, responded to >50% of calls, FGD 6]

**Prompt response to symptom reporting**

- *I personally think they should respond on that very same day that you report the symptom; so that you get help...sometimes you may be in great pain and you need urgent help...Doctor I would want to get my response as soon as I report the symptom*. [IDI Mulago, responded to 25%-50% of calls, FGD 4]

**Preference for two-way communication**

- *I would want to speak directly to the doctor...The doctor will speak to you, and you can inquire about anything. But as for the answering system, it will just do the talking but you won’t be able to talk back; all you do is just listen*. [KSG HCIV, responded to <25% of calls, FGD 1]

**Call for Life Uganda should have a separate space for its patients (clients) at health facilities**

- *As for me what I am saying; us the ones part of CFL, we should have our own space where we get our medicine, and also bring others to join which means we will be spreading CFL to our peers...so that means you can slowly by slowly bring other patients to join the system. If I bring some, and someone else brings in another, the CFL circle will get bigger*. [KSG HCIV, responded to <25% of calls, FGD 1]

## Discussion

### Principal Findings

This study complements the existing body of literature related to use and outcomes of mHealth communication technologies in health care delivery [30,42]. This substudy also complements our parent study that was conducted to determine the impact of IVR technology on Medical Outcomes HIV Quality of Life scores and viral suppression at 12 months, which showed better study outcomes and higher quality-of-life scores for participants with higher use of the CFLU tool than for low users [45]. It further complements another study (qualitative) by the team that explored the acceptability of a mobile phone support tool (CLFU) for promoting adherence to ART among young adults in a substudy of the RCT [48]. However, our study explored similarities and differences in the experiences, perceptions, and attitudes of people living with HIV regarding the CFLU tool among 3 categories based on the proportion of calls responded to. The study revealed that there were more similarities than differences in patients’ experiences with the CFLU tool. In particular, there was consensus across all groups that they had more positive experiences than negative ones with the CFLU system. However, participants who responded to >50% of calls reported more frequent use of the specific elements of the CFLU tool; hardly complained about the timing of the phone calls; and, consequently, experienced more benefits from the system than those who responded to calls <25% and between 25% and 50% of the time. This finding concurs with the results of the parent study, which revealed that, among participants in the intervention arm who were active at 12 months and had higher use of the CFLU tool than low users, there was a trend toward better study outcomes [45]. The challenges faced by the 2 categories who responded to <50% of the calls (mainly repeated challenges with the secret PIN code) are reflected in findings from the parent study and of another study that examined the feasibility of using IVR and SMS for automated collection of weekly individual-level ART adherence data in (rural) Southwestern Uganda [52]. In that study (which involved caregivers of children on ART who owned phones that were used to collect adherence data), Haberer et al [25,52] reported similar challenges, especially with the PIN, which reportedly caused most confusion, where >66% of the Ugandan patients with HIV studied were unable to use their mobile phone PINs to feed back information to the health care provider. Despite these challenges, the study recommended that the use of IVR and SMS in resource-limited settings is technically feasible. Suggestions made to improve response rates to address the aforementioned challenges were found applicable to our study as well, which included repeated trainings over time, training in groups so shy participants can learn from each other, and testing knowledge from the trainings.

Generally, participants across the 3 categories valued the confidentiality associated with the secret PIN code, privacy (place of choice), convenience (individual time), and comfort of using the CFLU tool provided. Given that HIV-related stigma is still prevalent, CFLU was generally acceptable as it provided participants with privacy. A similar observation was reported by Adeagbo et al [13,53], who found that mobile phone–connected HIV testing and web-based clinical care and prevention have the potential to support access to these services, particularly for young people and men whose uptake of these services remains low, especially in Africa.

A key finding is that our study unveiled that the 2 most popular application elements were pill reminders and health promotion tips. Irrespective of calls responded to, all participants largely attributed improved quality of life to pill reminders, followed by health promotion tips. Self-reported compliance to treatment resulting from pill reminders was a topic that ran across the 3 categories and was even more pronounced among participants who responded to <25% of the calls as most of them used the system solely as a pill reminder. Our findings are broadly consistent with those from a study conducted in 2 rural Ugandan districts, where adherence levels were significantly higher during mobile phone intervention [6]. Similar findings were reported in Kenya, where 2 RCTs sent ART reminders to people living with HIV, whose adherence reportedly improved significantly [28,29]. In addition, our results correspond to the findings from reviews and other studies that include both LMICs and the global north, where mHealth communication technologies improved adherence through SMS text messages and voice pill reminders [3,14,30].

Furthermore, participants who responded to >50% of calls listened more to health tips than their counterparts in the <25% and 25% to 50% FGDs and ably reported detailed health information that they had put into practice. Our findings are in agreement with other studies, which revealed that mHealth technologies are highly effective for the dissemination of health promotion messages such as nutrition advice and other lifestyle tips [19,33,35] and directly increased disease awareness with regard to transmission, prevention, treatment, and care among study participants (people living with HIV), who, in turn, reported that they sensitized their families and community members. Mobile phones are being increasingly used worldwide in health promotion and health care [19], where improvement in communication and information delivery was observed through symptom reporting and appointment reminders for clinic attendance [37]. However, contrary to this finding, a pilot study that explored the efficacy of an mHealth campaign using SMS as a platform to disseminate and measure HIV and AIDS knowledge and promote HIV and AIDS testing at clinics in rural Northwest Uganda had proportionately limited success in increasing knowledge levels on a mass scale [54]. Despite this challenge, the study recognized the potential of mHealth tools when extended to large numbers of mobile phone users as part of an integrated health campaign approach and suggests that mHealth campaigns need to be combined with other forms of dissemination in low-income countries where mobile phone access and literacy disparities exist.

Another discussion point is that most of the participants had good therapeutic relationships with the health care providers (eg, physicians, nurses, and counselors), which boosted their self-esteem, confidence, and trust in them and was a source of psychosocial support. Odili et al [10] believe that patient-care provider relationships and trust in the provider could be motivating factors for adherence.

An attempt has been made to provide concrete ideas on how to improve aspects of mHealth interventions in LMIC settings. As observed from the study findings, participants attributed their improved adherence, better health, and improved quality of life to CFLU and recommended that the tool be scaled to rural health facilities, nonparticipants, social places, and the media. Benefits of mHealth tools have been reported by other studies; for example, a systematic review of sociotechnical factors affecting patients’ adoption of mHealth tools revealed that mHealth adoption may improve health outcomes [55]. This review further explained that patients who perceive potential benefits such as better health effects and enhanced health behaviors from the use of the tools are more likely to use them.

Among the recommendations to improve aspects of mHealth tools is addressing technical challenges that reportedly affect mHealth tool performance, such as those mentioned in our study. This is because technical difficulties have been frequently cited as barriers, creating frustration and discouraging the embracing of mHealth tools [56]. Ease of use has been quoted as one of the leading factors affecting mHealth acceptance [57] and, if made possible, can greatly improve the usability of the tools in LMICs.

Training involving a more participatory approach is recommended given the different sociodemographic characteristics of patients, especially those in the older age groups and those with low levels of education that are common in LMICs [58]. In addition, provision of timely and adequate technical support may help users overcome technical challenges that were reportedly common among the study participants [59]. Coupled with the aforementioned issues, follow-up or continuous monitoring of the users by the health care providers may help provide feedback on use and challenges that can improve the performance of the different aspects of the mHealth tools [60].

Furthermore, studies have pointed out the importance of combining web-based and traditional health care provider communication to enable quicker and easier exchange of information between health care providers and patients [55,61,62]. Preference for two-way communication was also suggested by study participants.

Finally, provision of relevant and up-to-date and appropriate health education tailored to patients’ needs may greatly improve mHealth tools in LMICs where access to health education is constrained [56]. This may address knowledge gaps, raise disease awareness, and encourage healthier behaviors, consequently helping patients better understand their medications and possible side effects and symptoms and achieve better health results [55,56].

### Study Limitations

A potential limitation of this study is the fact that it was conducted in a setting where poor mobile phone coverage and frequent power and mobile phone network outages exist and loss of mobile phones is a common phenomenon. These findings may not be applicable to higher-income settings as such challenges would have implications in terms of sustainability of such an intervention. However, the findings of our study are not intended to be generalized to other settings. The resonance of our findings with other studies suggests that the findings may be applicable beyond the study area. Finally, the data in our study were based on informant responses, which might be subject to social desirability.

### Conclusions

In conclusion, our findings showed participants’ appreciation, high willingness, and interest in CFLU that demonstrated great potential to improve access to health care; adherence to treatment; health awareness; and, consequently, quality of life. The technology was well received, but the use of PIN codes for confidentiality was a challenge, and other confidentiality checks should be considered in our environment.

## Abbreviations

ART: antiretroviral therapy
CFLU: Call for Life Uganda
FGD: focus group discussion
IDI: Infectious Diseases Institute
IVR: interactive voice response
KSG HCIV: Kasangati Health Centre IV
LMIC: low- and middle-income country
mHealth: mobile health
PIN: personal identification number
RCT: randomized controlled trial

## References

[1] HIV/AIDS. World Health Organization.

[2] WHO Uganda Annual Report for 2018. World Health Organization.

[3] Finitsis DJ, Pellowski JA, Johnson BT (2014). Text message intervention designs to promote adherence to antiretroviral therapy (ART): a meta-analysis of randomized controlled trials. PLoS One.

[4] (2018). Uganda AIDS country progress report July 2017-June 2018. Government of Uganda.

[5] (2022). Joint United Nations Programme on HIV/AIDS (UNAIDS) Special Analysis 2019. unaids.org.

[6] Kunutsor S, Walley J, Katabira E, Muchuro S, Balidawa H, Namagala E, Ikoona E (2010). Using mobile phones to improve clinic attendance amongst an antiretroviral treatment cohort in rural Uganda: a cross-sectional and prospective study. AIDS Behav.

[7] Venter W, Coleman J, Chan VL, Shubber Z, Phatsoane M, Gorgens M, Stewart-Isherwood L, Carmona S, Fraser-Hurt N (2018). Improving linkage to HIV care through mobile phone apps: randomized controlled trial. JMIR Mhealth Uhealth.

[8] Rosen S, Fox MP (2011). Retention in HIV care between testing and treatment in sub-Saharan Africa: a systematic review. PLoS Med.

[9] Fox M, Rosen S (2010). Patient retention in antiretroviral therapy programs up to three years on treatment in sub-Saharan Africa, 2007-2009: systematic review. Trop Med Int Health.

[10] Odili VU, Obieche AO, Amibor KC (2017). Adherence to antiretroviral therapy and its determinants among HIV-infected patients in Nigeria. J Pharm Pract.

[11] Heestermans T, Browne JL, Aitken SC, Vervoort SC, Klipstein-Grobusch K (2016). Determinants of adherence to antiretroviral therapy among HIV-positive adults in sub-Saharan Africa: a systematic review. BMJ Glob Health.

[12] Reda AA, Biadgilign S (2012). Determinants of adherence to antiretroviral therapy among HIV-infected patients in Africa. AIDS Res Treat.

[13] Adeagbo O, Herbst C, Blandford A, McKendry R, Estcourt C, Seeley J, Shahmanesh M (2019). Exploring people's candidacy for mobile health-supported HIV testing and care services in rural KwaZulu-Natal, South Africa: qualitative study. J Med Internet Res.

[14] Kannisto KA, Koivunen MH, Välimäki MA (2014). Use of mobile phone text message reminders in health care services: a narrative literature review. J Med Internet Res.

[15] Chib A, van Velthoven MH, Car J (2015). mHealth adoption in low-resource environments: a review of the use of mobile healthcare in developing countries. J Health Commun.

[16] (2011). MHealth New Horizons for Health Through Mobile Technologies.

[17] (2016). Consolidated Guidelines on the Use of Antiretroviral Drugs for Treating and Preventing HIV Infection Recommendations for a Public Health Approach.

[18] Marent B, Henwood F, Darking M, EmERGE Consortium (2018). Development of an mHealth platform for HIV care: gathering user perspectives through co-design workshops and interviews. JMIR Mhealth Uhealth.

[19] Fischer H, Moore SL, Ginosar D, Davidson AJ, Rice-Peterson CM, Durfee MJ, MacKenzie TD, Estacio RO, Steele AW (2012). Care by cell phone: text messaging for chronic disease management. Am J Manag Care.

[20] Kruse C, Betancourt J, Ortiz S, Valdes Luna SM, Bamrah IK, Segovia N (2019). Barriers to the use of mobile health in improving health outcomes in developing countries: systematic review. J Med Internet Res.

[21] Kahn JG, Yang JS, Kahn JS (2010). 'Mobile' health needs and opportunities in developing countries. Health Aff (Millwood).

[22] Tamrat T, Kachnowski S (2012). Special delivery: an analysis of mHealth in maternal and newborn health programs and their outcomes around the world. Matern Child Health J.

[23] Chan CV, Kaufman DR (2010). A technology selection framework for supporting delivery of patient-oriented health interventions in developing countries. J Biomed Inform.

[24] Déglise C, Suggs LS, Odermatt P (2012). SMS for disease control in developing countries: a systematic review of mobile health applications. J Telemed Telecare.

[25] Bastawrous A, Armstrong MJ (2013). Mobile health use in low- and high-income countries: an overview of the peer-reviewed literature. J R Soc Med.

[26] Vervloet M, Linn AJ, van Weert JC, de Bakker DH, Bouvy ML, van Dijk L (2012). The effectiveness of interventions using electronic reminders to improve adherence to chronic medication: a systematic review of the literature. J Am Med Inform Assoc.

[27] Georgette N, Siedner MJ, Petty CR, Zanoni BC, Carpenter S, Haberer JE (2017). Impact of a clinical program using weekly Short Message Service (SMS) on antiretroviral therapy adherence support in South Africa: a retrospective cohort study. BMC Med Inform Decis Mak.

[28] Lester RT, Ritvo P, Mills EJ, Kariri A, Karanja S, Chung MH, Jack W, Habyarimana J, Sadatsafavi M, Najafzadeh M, Marra CA, Estambale B, Ngugi E, Ball TB, Thabane L, Gelmon LJ, Kimani J, Ackers M, Plummer FA (2010). Effects of a mobile phone short message service on antiretroviral treatment adherence in Kenya (WelTel Kenya1): a randomised trial. Lancet.

[29] Pop-Eleches C, Thirumurthy H, Habyarimana J, Zivin J, Goldstein M, de Walque D, MacKeen L, Haberer J, Kimaiyo S, Sidle J, Ngare D, Bangsberg DR (2011). Mobile phone technologies improve adherence to antiretroviral treatment in a resource-limited setting: a randomized controlled trial of text message reminders. AIDS.

[30] Mbuagbaw L, Mursleen S, Lytvyn L, Smieja M, Dolovich L, Thabane L (2015). Mobile phone text messaging interventions for HIV and other chronic diseases: an overview of systematic reviews and framework for evidence transfer. BMC Health Serv Res.

[31] Mbuagbaw L, van der Kop ML, Lester RT, Thirumurthy H, Pop-Eleches C, Ye C, Smieja M, Dolovich L, Mills EJ, Thabane L (2013). Mobile phone text messages for improving adherence to antiretroviral therapy (ART): an individual patient data meta-analysis of randomised trials. BMJ Open.

[32] Crankshaw T, Corless IB, Giddy J, Nicholas PK, Eichbaum Q, Butler LM (2010). Exploring the patterns of use and the feasibility of using cellular phones for clinic appointment reminders and adherence messages in an antiretroviral treatment clinic, Durban, South Africa. AIDS Patient Care STDS.

[33] Littman-Quinn R, Chandra A, Schwartz A, Chang A, Fadlelmola F, Ghose S, Armstrong K, Bewlay L, Digovich K, Seymour SK, Kovarik CL (2011). mHealth applications for clinical education, decision making, and patient adherence in Botswana. Proceedings of the 2011 IST-Africa Conference Proceedings.

[34] Rana Y, Haberer J, Huang H, Kambugu A, Mukasa B, Thirumurthy H, Wabukala P, Wagner GJ, Linnemayr S (2015). Short message service (SMS)-based intervention to improve treatment adherence among HIV-positive youth in Uganda: focus group findings. PLoS One.

[35] John M, Samson-Akpan P, Etowa J, Akpabio I, John E (2016). Enhancing self-care, adjustment and engagement through mobile phones in youth with HIV. Int Nurs Rev.

[36] Head KJ, Noar SM, Iannarino NT, Grant Harrington N (2013). Efficacy of text messaging-based interventions for health promotion: a meta-analysis. Soc Sci Med.

[37] Car J, Gurol‐Urganci I, de Jongh T, Vodopivec‐Jamsek V, Atun R (2012). Mobile phone messaging reminders for attendance at healthcare appointments. Cochrane Database of Systematic Reviews.

[38] Guy R, Hocking J, Wand H, Stott S, Ali H, Kaldor J (2012). How effective are short message service reminders at increasing clinic attendance? A meta-analysis and systematic review. Health Serv Res.

[39] Berger E (2010). Telemedicine: has its time come?. Ann Emerg Med.

[40] Blake H (2008). Innovation in practice: mobile phone technology in patient care. Br J Community Nurs.

[41] Mechael P (2010). Barriers and gaps affecting mHealth in low and middle income countries : policy white paper. Columbia university.

[42] Abaza H, Marschollek M (2018). mHealth application areas and technology combinations. Methods Inf Med.

[43] Qiao S, Zhang Y, Li X, Menon JA (2018). Facilitators and barriers for HIV-testing in Zambia: a systematic review of multi-level factors. PLoS One.

[44] de Tolly K, Skinner D, Nembaware V, Benjamin P (2012). Investigation into the use of short message services to expand uptake of human immunodeficiency virus testing, and whether content and dosage have impact. Telemed J E Health.

[45] Byonanebye DM, Nabaggala MS, Naggirinya AB, Lamorde M, Oseku E, King R, Owarwo N, Laker E, Orama R, Castelnuovo B, Kiragga A, Parkes-Ratanshi R (2021). An interactive voice response software to improve the quality of life of people living with HIV in Uganda: randomized controlled trial. JMIR Mhealth Uhealth.

[46] Macleod B, Phillips J, Stone A, Walji A, Awoonor-Williams JK (2012). The architecture of a software system for supporting community-based primary health care with mobile technology: the mobile technology for community health (MoTech) initiative in Ghana. Online J Public Health Inform.

[47] Swendeman D, Jana S, Ray P, Mindry D, Das M, Bhakta B (2015). Development and pilot testing of daily interactive voice response (IVR) calls to support antiretroviral adherence in India: a mixed-methods pilot study. AIDS Behav.

[48] Twimukye A, Bwanika Naggirinya A, Parkes-Ratanshi R, Kasirye R, Kiragga A, Castelnuovo B, Wasswa J, Nabaggala MS, Katabira E, Lamorde M, King RL (2021). Acceptability of a mobile phone support tool (call for life Uganda) for promoting adherence to antiretroviral therapy among young adults in a randomized controlled trial: exploratory qualitative study. JMIR Mhealth Uhealth.

[49] Download NVivo. NVivo.

[50] Braun V, Clarke V (2006). Using thematic analysis in psychology. Qual Res Psychol.

[51] Baxter P, Jack S (2015). Qualitative case study methodology: study design and implementation for novice researchers. Qual Report.

[52] Haberer JE, Kiwanuka J, Nansera D, Wilson IB, Bangsberg DR (2010). Challenges in using mobile phones for collection of antiretroviral therapy adherence data in a resource-limited setting. AIDS Behav.

[53] Siu GE, Wight D, Seeley JA (2014). Masculinity, social context and HIV testing: an ethnographic study of men in Busia district, rural eastern Uganda. BMC Public Health.

[54] Chib A, Wilkin H, Ling LX, Hoefman B, Van Biejma H (2012). You have an important message! Evaluating the effectiveness of a text message HIV/AIDS campaign in Northwest Uganda. J Health Commun.

[55] Jacob C, Sezgin E, Sanchez-Vazquez A, Ivory C (2022). Sociotechnical factors affecting patients' adoption of mobile health tools: systematic literature review and narrative synthesis. JMIR Mhealth Uhealth.

[56] Vo V, Auroy L, Sarradon-Eck A (2019). Patients' perceptions of mHealth apps: meta-ethnographic review of qualitative studies. JMIR Mhealth Uhealth.

[57] Salgado T, Tavares J, Oliveira T (2020). Drivers of mobile health acceptance and use from the patient perspective: survey study and quantitative model development. JMIR Mhealth Uhealth.

[58] De La Cruz Monroy MF, Mosahebi A (2019). The use of smartphone applications (apps) for enhancing communication with surgical patients: a systematic review of the literature. Surg Innov.

[59] Abelson JS, Kaufman E, Symer M, Peters A, Charlson M, Yeo H (2017). Barriers and benefits to using mobile health technology after operation: a qualitative study. Surgery.

[60] Li J, Varnfield M, Jayasena R, Celler B (2021). Home telemonitoring for chronic disease management: perceptions of users and factors influencing adoption. Health Informatics J.

[61] Runz-Jørgensen SM, Schiøtz ML, Christensen U (2017). Perceived value of eHealth among people living with multimorbidity: a qualitative study. J Comorb.

[62] Lie SS, Karlsen B, Oord ER, Graue M, Oftedal B (2017). Dropout from an eHealth intervention for adults with type 2 diabetes: a qualitative study. J Med Internet Res.

